# Dilated Cardiomyopathy and Nail-Patella Syndrome: A Case Report

**DOI:** 10.7759/cureus.48969

**Published:** 2023-11-17

**Authors:** Nasatya Khadka, Pooja KC, Shailendra Sharma, Sandesh Sharma, Sumugdha Rayamajhi

**Affiliations:** 1 Medicine, Emilio Aguinaldo College, Manila, PHL; 2 Internal Medicine, Nepal Development Society, Kathmandu, NPL; 3 Nephrology, Sparrow Health System, Lansing, USA; 4 Internal Medicine, Kausilya Aspatal, Banke, NPL; 5 Internal Medicine, Michigan State University College of Human Medicine, East Lansing, USA

**Keywords:** nail-patella syndrome, dysplastic fingernail, dysplastic patella, pitted nail, end stage renal disease (esrd), dilated cardiomyopathy (dcm)

## Abstract

Nail-patella syndrome (NPS) is a rare genetic disorder with multiple skeletal deformities and a variety of extra-skeletal involvements. We present a 17-year-old male with a clinical tetrad of skeletal abnormalities, multiple bony deformities, advanced renal failure, hypothyroidism, and dilated cardiomyopathy. A clinical diagnosis of NPS was made, supported by radiographic findings, and corroborated by compatible renal biopsy results. There are very few published reports describing the association of dilated cardiomyopathy with this syndrome. A high index of suspicion is needed to make this diagnosis, given myriads of multi-systemic manifestations.

## Introduction

Nail-patella syndrome (NPS) is an autosomal dominant disorder with classic skeletal features of dysplastic fingernails, hypoplastic or aplastic patella, presence of iliac horns, and elbow deformities. It has an estimated incidence of 1 per 50,000 individuals and results from loss-of-function mutations in the LMX1B gene, with more than 140 different mutations identified to date [1]. Aside from classic skeletal features, it has a wide range of systemic involvements with renal, cardiac, ocular, and neurological manifestations. Renal involvement is a major pathological feature that is common and contributes significantly to overall morbidity and mortality. Manifestations range from mild renal abnormalities to advanced chronic kidney disease and end-stage renal disease. The diagnosis of NPS is clinical, with the characteristic physical exam and radiological imaging findings confirmed by genetic testing and renal biopsy. Our patient had classic skeletal features and a multitude of symptoms known to be associated with NPS.

## Case presentation

**Figure 1:**
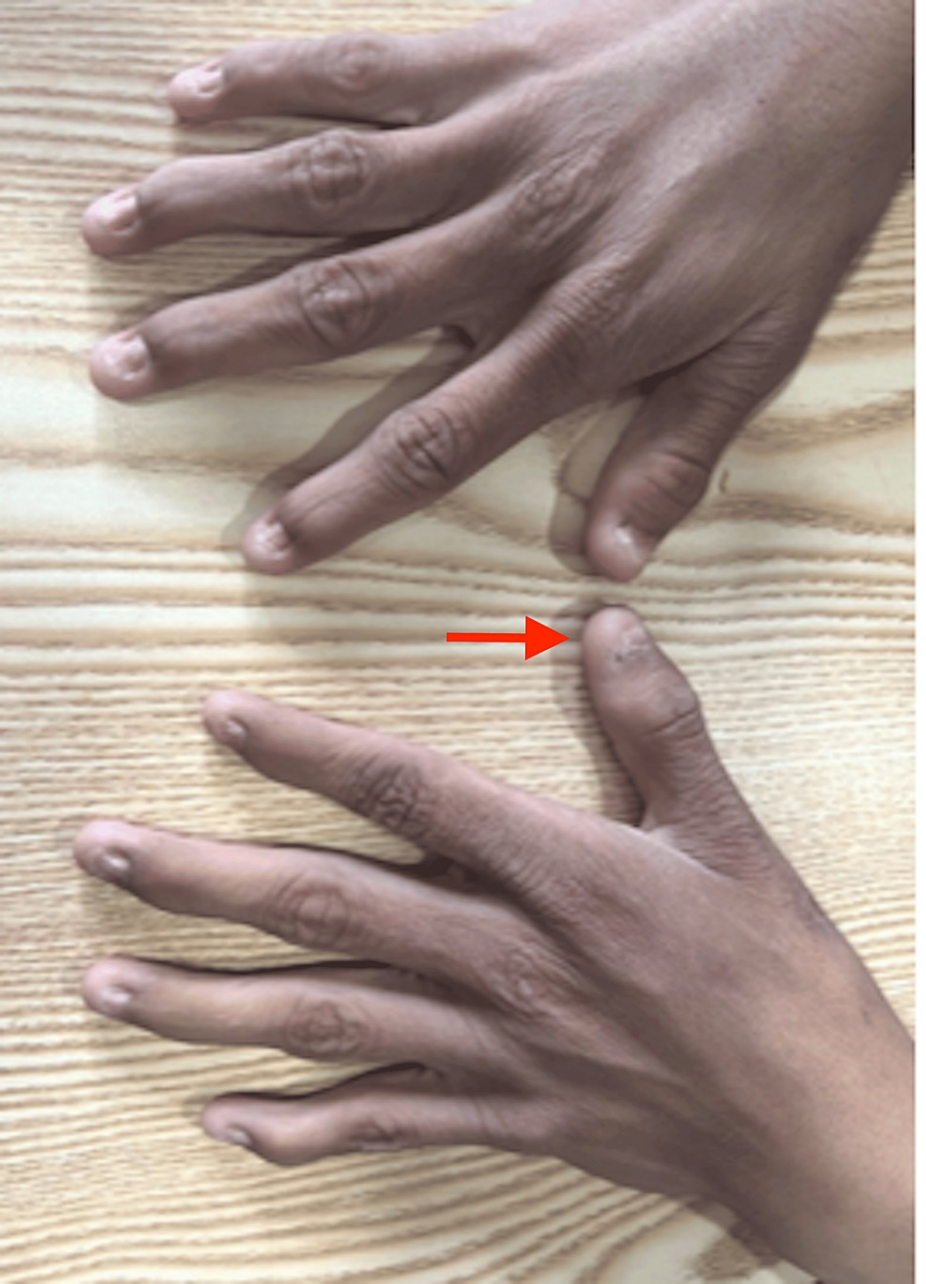
Bilateral hands showing dysmorphic and dysplastic fingernails

**Figure 2:**
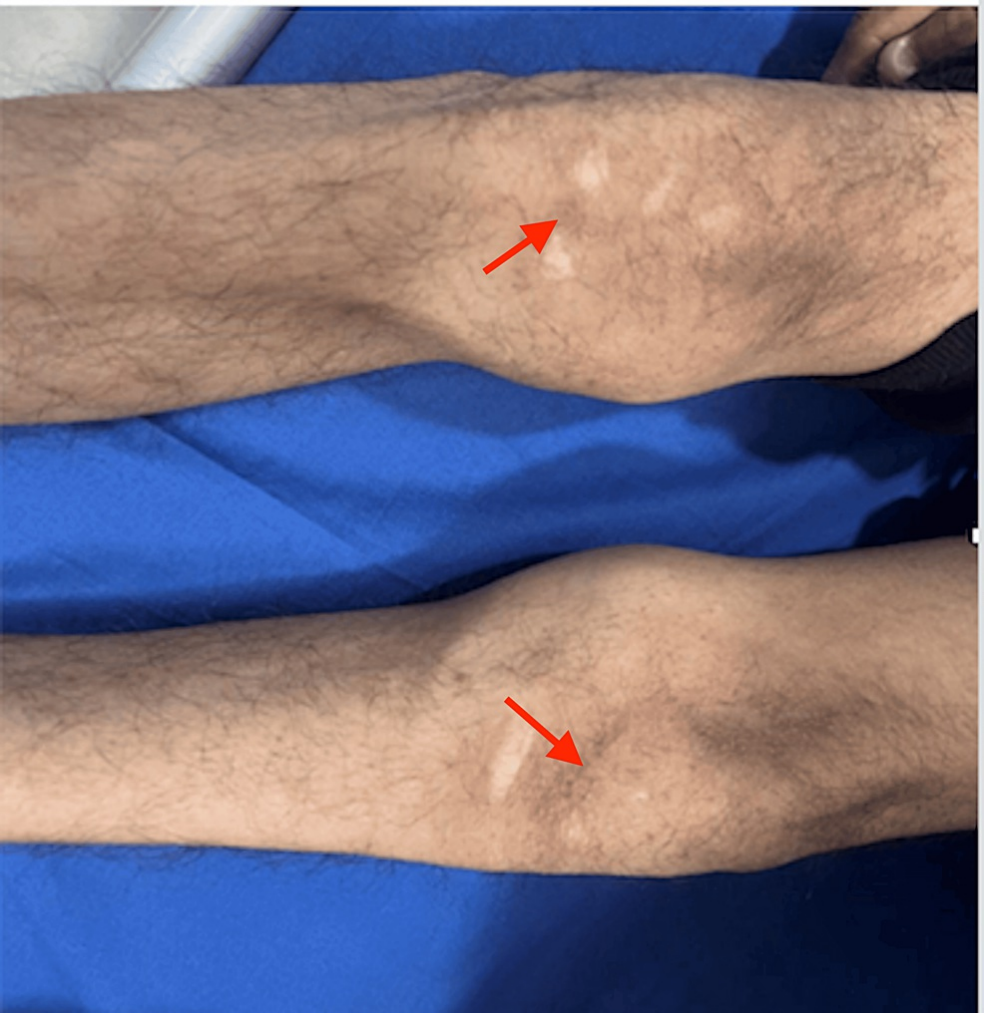
Deformed bilateral knee joints with patellar hypoplasia

A 17-year-old male presented with a one-month history of nausea, anorexia, abdominal discomfort, intermittent diarrhea, and abdominal pain with cramps. He reported a gradual decline in muscle mass, thinning, and atrophy, specifically affecting the left lower limb. Subsequently, he developed poor urine output, shortness of breath, facial puffiness, and swelling of the legs. Past surgical history was significant for surgery on the left foot for club foot at the age of two years. Physical examination revealed conjunctival pallor, bilateral pitting pedal edema, and dystrophic and dysplastic nails affecting all fingers (Figure 1) and toes, with the thumb exhibiting more prominent deformities. Extremity examination showed deformed bilateral knee joints with patellar hypoplasia (Figure 2), fixed flexion deformity of bilateral arms, and adduction, equines, and cavus deformity of the left foot. Chest auscultation revealed fine bibasal crepitations. There was a soft systolic murmur over the pulmonary area and no pericardial rub. Examination of the abdomen, central nervous system (CNS), and fundus was normal.

**Figure 3:**
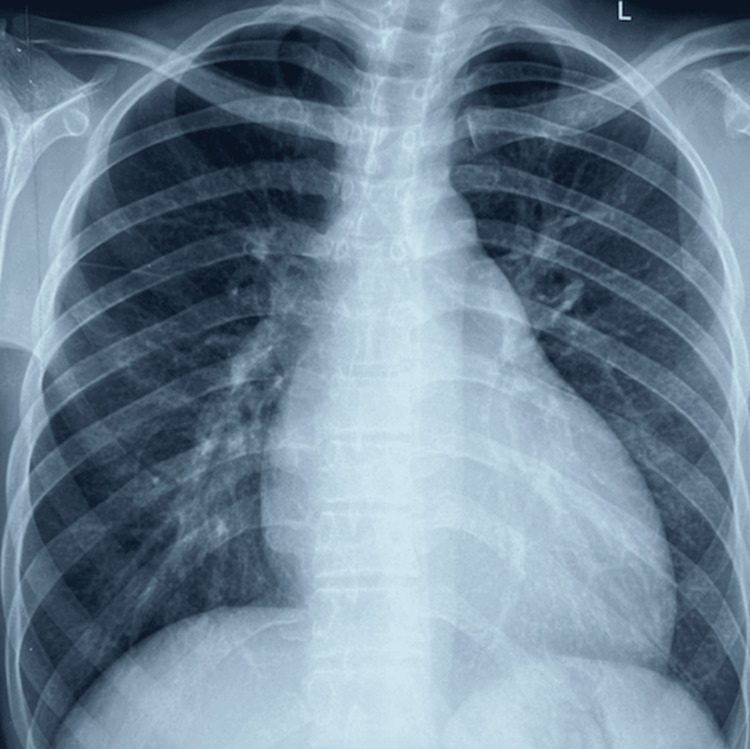
Chest X-ray with mild cardiomegaly

**Figure 4:**
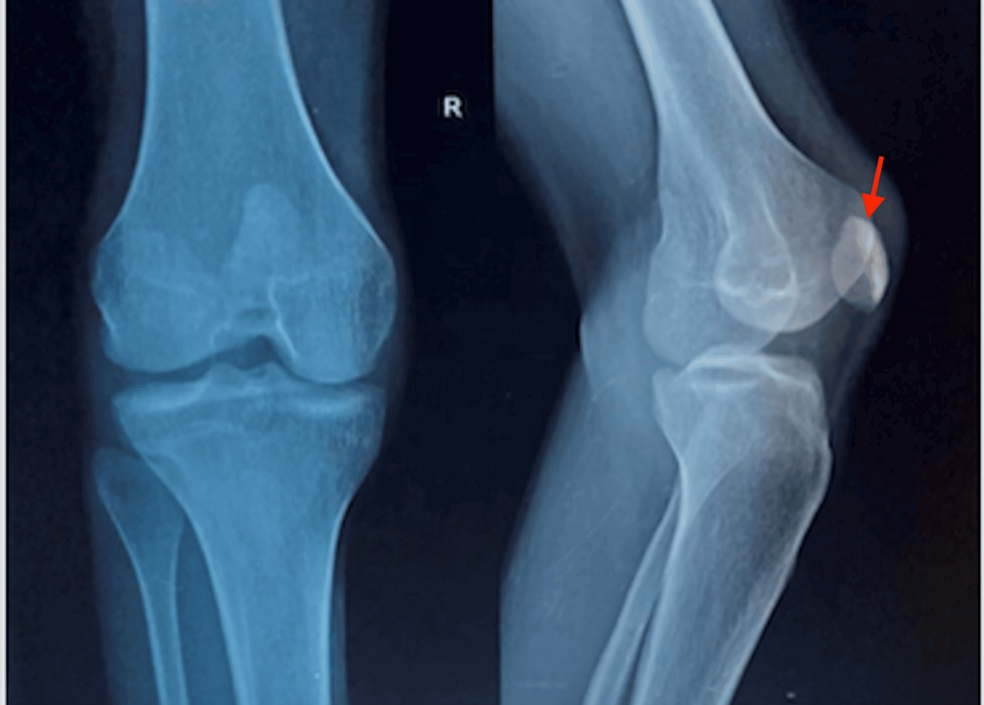
Bilateral knee X-ray showing hypoplastic and dysplastic patella

**Figure 5:**
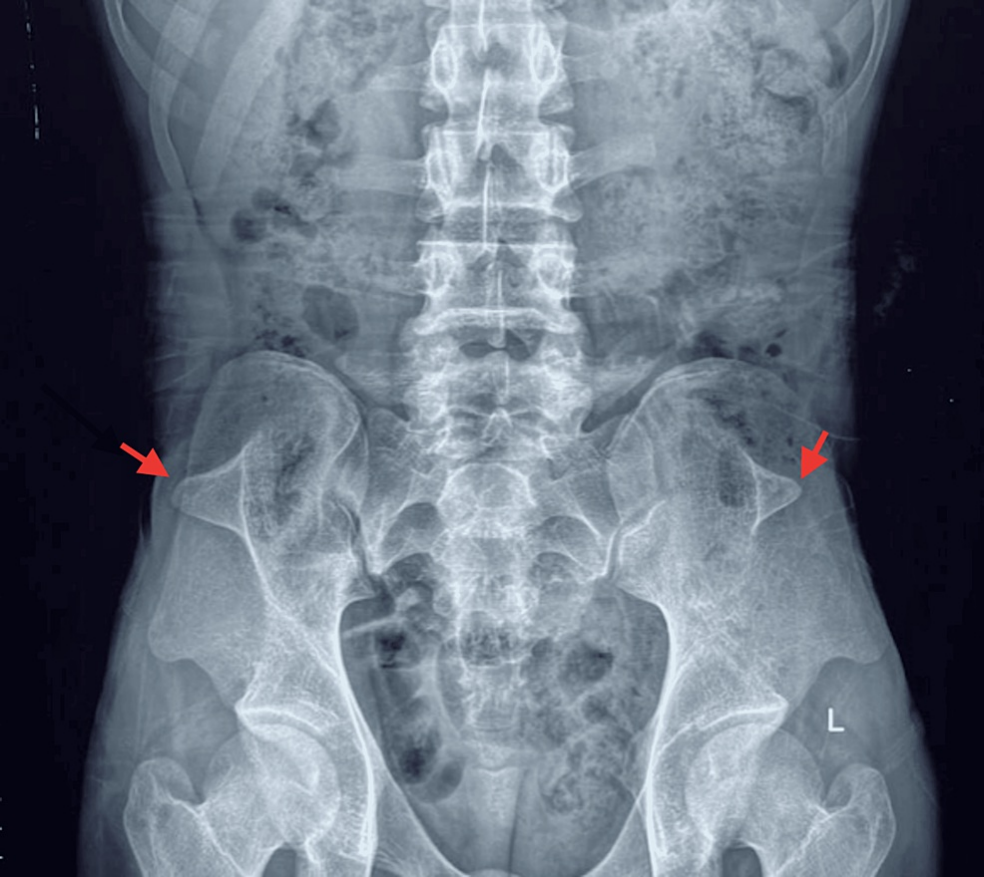
Pelvic X-ray showing bilateral iliac horns

Labs show elevated blood urea nitrogen (BUN) 16.8 mmol/L and creatinine 426 µmol/L, high thyroid-stimulating hormone (TSH) 6.14 m IU/L (reference range 0.5-4.5 m IU/L), with low T4 and normal T3 levels. Urinalysis was negative for blood, casts, and cells. Twenty-four-hour urine collection showed 7300 gm of protein. Chest X-ray showed mild cardiomegaly (Figure 3). A transthoracic echocardiogram (TTE) showed ejection fraction of 40%, left ventricular (LV) global hypokinesia, mild pericardial effusion (anterior: 2 mm, posterior: 6 mm, lateral: 5 mm), mild mitral regurgitation, mild pulmonary regurgitation, mild tricuspid regurgitation (peak gradient: 40 mmHg), dilated left atrium (LA) and left ventricle (LV). Renal ultrasound showed right kidney measuring 10.7 cm x 3.8 cm, left kidney measuring 9.7 cm x 5.3 cm, bilateral increased echotexture with grade II medullary nephrocalcinosis. X-ray of knee joints exhibited dysplastic patellae (Figure 4), and pelvic X-ray revealed bilateral iliac horns (Figure 5). Renal biopsy showed global tuft sclerosis in 3 out of 15 glomeruli, segmental to near-complete sclerosis in the remaining tufts, accompanied by prominent intraglomerular foam cell changes.

We made a diagnosis of nail-patella syndrome based on the comprehensive clinical presentation, severe renal involvement (with nephrotic-range proteinuria, severe chronic kidney disease) and classic clinical tetrad of dysplastic fingernails, hypoplastic or aplastic patella, presence of iliac horns and elbow deformities. He was started on hemodialysis via a central venous catheter, and a brachiocephalic fistula surgically created on the left arm for more stable vascular access.

## Discussion

Nail-patella syndrome is a rare autosomal dominant disease with a myriad of skeletal and extraskeletal abnormalities. Characteristic features of this syndrome are a variety of bone and nail abnormalities. Our patient had multiple physical examination findings suggesting NPS. The patient appears thin and frail with a body mass index of 13.3 kg/m2, and had prominent bony structures throughout his body; he displayed dystrophic and dysplastic pitted nails with loss of skin creases in the distal interphalangeal joint and a "swan neck deformity" in the fingers. The severity of nail features intensified from the fifth finger to the thumb. Visible elbow deformity with limited range of motion was also observed. Given the rarity of nail-patella syndrome, encounters with affected individuals are infrequent, in resource-limited settings, the diagnosis and management of such rare diseases can pose challenges due to limited access to comprehensive investigations. Consequently, prompt identification of the syndrome may be delayed, as was the case with our patient, who was diagnosed after developing symptoms of renal failure, which rapidly progressed to end-stage renal disease.

The kidney biopsy demonstrated the presence of widespread global and segmental sclerosing lesions with findings suggestive of tubulointerstitial chronicity. These findings are consistent with those reported in the existing literature [2,3]. Subsequent routine investigations revealed that the patient had hypothyroidism and dilated cardiomyopathy. An extensive literature search led to the identification of two case reports discussing the association between nail-patella syndrome and thyroid disorders. The case report by Haras et al. described a case of hyperthyroidism, where the author suggested that the finding might be coincidental [4], and the case report by Goecke documented hypothyroidism in nail-patella syndrome [5]. Previous genetic studies have identified that the LMX1B gene product interacts with cofactor of LIM (CLIM) protein. These CLIM proteins are expressed in thyroid tissue, and since the LMX1B gene is implicated in nail-patella syndrome, it would be speculative to establish a link between NPS and thyroid-related conditions without further context and research [6,7]. It is noteworthy that thyroid disorders and cardiomyopathies can coexist with chronic kidney disease [8,9]. The patient had dilated cardiomyopathy with depressed ejection fraction (EF) and global hypokinesia on TTE. There are no published reports/ articles associating cardiomyopathies with nail-patella syndrome. 

It is noteworthy that club foot deformities can be frequently associated with NPS [1]. Our patient underwent open reduction and internal fixation of left club foot deformity at the age of two. A study by Towers et al. showed that bone mineral density (BMD) can be as low as 20% of gender-matched adults, with the odds of prevalence of fracture being 30.9 among patients with NPS; hence, patients with NPS are associated with decreased BMD and increase susceptibility to fracture [10].

NPS patients frequently experience gastrointestinal symptoms such as loose stools, diarrhea or constipation, abdominal cramps etc. [11]. The patient in this case also reported a history of chronic gastrointestinal symptoms such as cramping abdominal pain associated with loose stool. Despite undergoing thorough investigation such as comprehensive blood, serological investigations, ultrasonography of the abdomen, and upper and lower gastrointestinal endoscopy, no discernible underlying pathology was detected, which helped make the diagnosis of nail-patella syndrome.

## Conclusions

Healthcare professionals should maintain a high index of suspicion for nail-patella syndrome when encountering patients with acute renal failure, low BMI, and bone and nail deformities. The association of dilated cardiomyopathy with this syndrome has not been described, and to our knowledge, this is the first case report of this. Increased awareness and interdisciplinary collaboration among clinicians, geneticists, and nephrologists are imperative for accurate diagnosis and comprehensive management, including genetic counseling, in individuals and their families affected by NPS. Further research is warranted to elucidate the precise mechanisms underlying the diverse clinical features of NPS and to explore potential associations with incidental findings, paving the way for better understanding and tailored interventions.
